# Does birth under-registration reduce childhood immunization? Evidence from the Dominican Republic

**DOI:** 10.1186/s13561-017-0149-3

**Published:** 2017-03-23

**Authors:** Steve Brito, Ana Corbacho, Rene Osorio

**Affiliations:** 1 International Monetary Fund, 700 19th St NW, Washington, DC 20431 USA; 2 Inter-American Development Bank, 1300 New York Avenue NW, Washington, DC 20577 USA

**Keywords:** Immunization, Under-registration, Health access

## Abstract

The consequences of lacking birth certificates remain largely unexplored in the economic literature. We intend to fill this knowledge gap studying the effect of lacking birth certificates on immunization of children in the Dominican Republic. This is an interesting country because a significant number of children of Haitian descent face the consequences of lacking proper documentation. We use the distance to the civil registry office and the mother’s document of identification as instrumental variables of the child’s birth certificate. After controlling for distance to immunization services and other determinants, this paper finds that children between 0 and 59 months of age that do not have birth certificates are behind by nearly one vaccine (out of a total of nine) compared to those that have birth certificates.

## Background

Birth registration, which provides legal proof of a child’s existence and nationality, is considered a fundamental human right according to the Convention on the Rights of the Child (1989). In many countries, identity documents are required to access benefits such as school diplomas, health care services, conditional cash transfers, pensions, banking services, civil rights, adoption, divorce, marriage and inheritance.

**Fig. 1:**
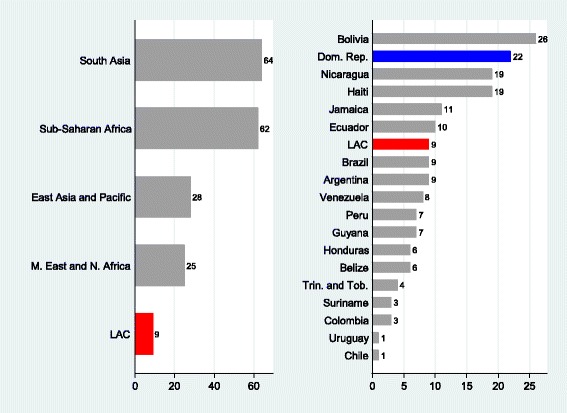
Percentage of children without birth certificates, Age 0–4, 2000–2010*

This paper to sheds light on the effect of birth under-registration on health access. Childhood immunizations, a key component of health care services, are intended to be administered to all children on a standardized schedule. The paper focuses on the effect of under-registration of births on childhood immunization in the Dominican Republic, the country with the second highest percentage (22%) of children under the age of 5 without birth certificates in Latin American and Caribbean (LAC) countries (UNICEF [39]) (Fig. 1).

Studying the factors that affect immunization in the region is important because proper vaccination can reduce infant morbidity (Aaby et al.[1]; Bishar et al. [10]; Breiman et al. [16]). Vaccination is also crucial in reducing infant mortality under 5, according to the fourth Millennium Development Goal (MDG4). Furthermore, vaccinating communities reduces the risk of disease outbreaks and their spread to neighboring communities. Many studies have shown that vaccination at the appropriate age has positive effects on cognitive development, educational achievement, and productivity in developing countries (Bloom et al. [12]; Canning et al. [17]).

Another reason to study immunization determinants is that increasing vaccination coverage is cost-effective. According to the World Health Organization (WHO), polio eradication saved governments US$1.5 billion per year in treatment and rehabilitation costs (Bloom et al. [11]). The Institute of Medicine reports that for every dollar spent on the MMR vaccine, US$21 is saved (Bloom et al.[11]). Extensive literature on the economics of immunization finds good reasons for vaccination due to its cost-benefit and/or cost-effectiveness (WHO [40]).

After controlling for well-established socioeconomic determinants of immunization and endogeneity, this study found that those children without birth certificates have 0.7 vaccines fewer than children with birth certificates. As variables were included that might be correlated to the instruments, the results were found to be robust to threats to the exclusion restriction of the instrumental variables such as location of immunization centers and mobile immunization campaigns.

The reason that undocumented children receive fewer vaccinations is because they cannot be registered in the Dominican social security system, which guarantees access to public vaccination facilities or reimburses costs incurred in private health facilities. Moreover, the lack of a birth certificate makes it difficult to prove age, and most countries, including the Dominican Republic, follow WHO’s immunization schedule, which is based on the age of the child. The two vaccines that have lower probability of being delivered are the first doses of polio (OPV1), and pertussis, tetanus, and diphtheria (DTP1). The result is reduced immunization rates and/or delays in vaccine administration.

The rest of the paper is organized as follows. Section 2 reviews the related literature and examines factors associated with the registration of children’s births and immunization. Section 3 presents the data used and the methodology and potential econometric difficulties, and Section 4 analyzes the results and provides conclusions.

## Literature review

Qualitative studies in the LAC region have shown that children without identity documents have more difficulty accessing public services, including health services. Bracamonte and Ordonez [15] cover the effects of the lack of a birth certificate in Chile, Colombia, Honduras, Ecuador, Nicaragua, and Peru on access to education, health services, and conditional cash transfers. Harbitz and Tamargo [32] explore the factors that contribute to under-registration of births and lack of legal identity. Harbitz and Boekle-Giuffrida [31] document the diverse challenges faced by those lacking legal identity documents. Cody [21] finds that birth registration is a prerequisite for accessing health services in many developing regions.

But the consequences of the lack of birth certificates are only beginning to be studied. In this regard, Castro and Rud [18] find a correlation between education and identity documents in children and adults. Brito et al. [23] study the effects of the lack of birth certificates on educational attainment and conclude that birth under-registration reduces educational attainment. Gine and Yang [29] link the development of fingerprinting in Malawi, a very accurate technology of personal identification, with improvements in borrowers’ creditworthiness, repayment rates, and expansion of the credit received. Fagernas [27] finds increased enforcement of child labor laws and educational attainment in the early 20th century in the United States, after birth registration laws were approved.

This paper examines the consequences of the lack of birth certificates on immunization. Immunization is studied rather than other health care services due to the availability of data, but other health-related programs, such as maternal care, may also be affected by the lack of a legal identity. Immunization programs have been more successful in reaching segments of the most disadvantaged populations in developing countries. In fact, according to WHO, by 2010, LAC countries had achieved coverage above 90% of measles vaccines (MCV) and the three recommended doses of DTP among children aged 12-23 months. Worldwide, coverage rates are typically above 75%, even in the least developed regions.

Notwithstanding these high coverage rates, they are not complete. The lack of services due to system failures, poor public awareness, and misconceptions even in well-developed countries are among the reasons behind incomplete immunization schedules (Schmitt [37]; Discover Magazine [24]). Other factors associated with under-immunization are race, ethnicity, birth order, marital status of the respondent, number of children in the household, access to public or private health insurance, decentralization of public services, and conditional cash transfers, among others (Adler et al. [3]; Feilden and Nielsen [28]; Barker et al. [8]; Khalegian [34]; Bardenheier et al. [6]; Chaui et al. [19]; Berman et al. [9]; Bakirci and Torun [5]; Acemoglu et al. [2]; Barham and Maluccio [7]).

Vaccine coverage is the most frequently used indicator of immunization among children between 12 and 23 months of age, but delays in delivery are overlooked (Chu et al. [20]; Faustini et al. [30]; Hull et al. [33]; Akmatov et al. [4]). Vaccines have the highest effectiveness during the recommended age range, and yet show lower compliance than uptake rates. Therefore, timely vaccination rather than coverage may be more important when the timing of delivery is crucial (Bolton et al. [14]). Factors affecting delay are similar to those affecting uptake. Single parenting, parental education, large family size, insurance coverage, and birth order have been documented as affecting delays in vaccination (Bobo et al. [13]; Essex et al. [26]; Dombkowski et al. [25]). This analysis encompasses both immunization coverage and timely vaccination.

## Data

The data come from the 2007 Demographic and Health Survey (DHS) of the Dominican Republic. The DHS includes extensive information on health and education outcomes, as well as household socioeconomic characteristics. It is among the few surveys with information on identity documents.^1^ The DHS of the Dominican Republic contains data on geographic location of clusters of households. The data collection was done in the year 2007 between March and August using face to face interviews completed in 97% of the houses selected of a total of 33,437. The questionnaire used during the interview was answered by mostly females (aged 15-19) although efforts were made to interview males (aged 15-59). This DHS survey was collected while the people were present in their homes. Hence, data from hospitals, health centers or immunization records did not form part of the survey to avoid problems with self-selection issues. The two other sources of data are global positioning system (GPS) data on civil registry offices and on immunization centers.

The main variables of interest are immunization outcomes. All children with complete or incomplete vaccinations and those who had never been vaccinated were included in the econometric analysis. Only those children who died before the data was collected are not part our sample. Of the 5,157 children in our sample 4% reported that a child had died, however we do not have information on the immunization records or birth registration of the deceased children. The survey does not contain information about the cause of death which might include unvaccinated children dying from diseases that vaccination might have prevented. For this reason, it is difficult to say anything about the type of bias or censoring this might introduce to our analysis. More problematic could be inaccurate recall of the immunization records of the children without immunization cards. For these children, their parents might have stated incorrectly the number of doses received of a given vaccine included, causing inconsistent estimations in our econometric analysis. Fortunately, the survey recorded information from immunization cards, which were available in 71% of the children in our sample. We used information from vaccination cards for a robustness check in Table 8.

The focus is on the nine vaccines recommended by the WHO in its extended program of immunization (EPI) worldwide. Hence, the vaccines analyzed in this paper are the following:

- one dose of Bacillus Calmette-Guerin (BCG) and one of hepatitis b (HEPB)
- three doses of Diphtheria Tetanus Pertussis (DTP) or three doses of pentavalent which includes five vaccines in one shot against diphtheria, tetanus, pertussis, hepatitis B and haemophilus influezae b
- three doses of Polio (OPV)
- one dose of measles (MCV) or one dose of triple viral containing vaccines against measles, mumps and rubella (MMR) in one shot

**Fig. 2:**
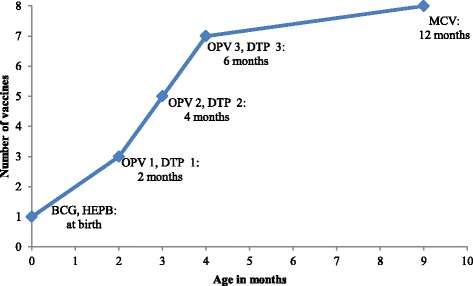
Vaccination schedule

Figure 2 shows the number of vaccines as well as the age range recommended to receive them.

During the survey, mothers were asked to show the vaccination card (70% compliance) to verify whether or not the children had been vaccinated. The cards also contained the day, month, and year of vaccination. Those mothers who reported not having their children’s vaccination card responded from memory but did not provide the date of vaccination. Some literature has found that data using parental recall slightly underreports immunization rates (see Simpson et al. [38]; Langsten and Hill [35]). To check the robustness of the results to this potential measurement error, we repeated the analysis described below with the subsample of children with vaccination cards and obtained similar results. Comparisons with the study’s main results are reported in the Appendix.

**Fig. 3:**
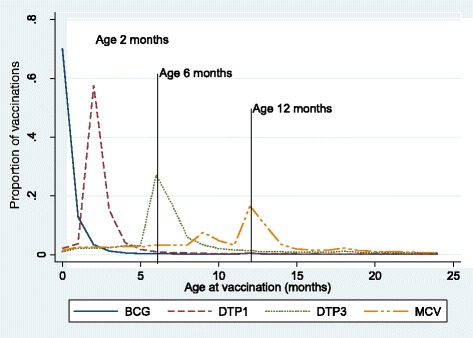
Dominican Republic: distribution of age by vaccines

The DHS 2007 contains information on the current age in months of the children at the moment of the interview. We used this information to construct Fig. 3. This figure shows the age distribution for administration of the BCG, DTP1, DTP3 and MCV vaccines. We excluded from the figure the age distribution corresponding to the HEPB and the OPV vaccines because the former is superimposed with the distribution of the BCG and the latter with those of the DTPs. All data in Fig. 3 are from the subsample with vaccination cards the delay in age-appropriate vaccination for those without cards cannot be calculated.

All distributions peak around the recommended age, indicating that most children receive their vaccines when they are due, but they also have long right-sided tails, indicating delays. On the other hand, shorter left tails suggest that premature delivery is less frequent. The distribution of the BCG that is administered after birth shows the least prominent tail, perhaps because it is delivered at birth. The distributions for those vaccines administered after birth show more significant delays.

Table 1 contains the summary statistics. The BCG, delivered usually at birth, shows the highest percentage of compliance (98%). This is consistent with the fact that 98% of the children are born in hospitals, health centers, or with medical attention. The data show that the administration of vaccines diminishes monotonically after birth, so the first vaccines have higher uptake rates, while the MCV, given at 9–12 months, has the lowest.

**Table 1:** Summary Statistics for the Dominican Republic 2007 for Children aged 0–59 Months

	(1.1)	(1.2)	(1.3)	(1.4)	(1.5)
Variables	N	mean	sd	min	max
*Dependent variables:*					
Number of vaccines	5,157	7.630	1.990	0	9
BCG uptake	5,157	0.984	0.127	0	1
HEPB uptake	5,157	0.943	0.232	0	1
DTP1 uptake	5,157	0.925	0.264	0	1
DTP2 uptake	5,157	0.843	0.364	0	1
DTP3 uptake	5,157	0.730	0.444	0	1
OPV1 uptake	5,157	0.948	0.222	0	1
OPV2 uptake	5,157	0.859	0.348	0	1
OPV3 uptake	5,157	0.699	0.459	0	1
MCV uptake	5,157	0.699	0.459	0	1
Proportion of age-due vaccines (age > 12 months)	3,478	0.589	0.291	0	1
Complete vaccination at 7 months of age	4,314	0.235	0.424	0	1
Complete vaccination at 13 months of age	4,315	0.231	0.422	0	1
*Endogenous variable:*					
Child without birth certificate	5,157	0.188	0.390	0	1
*Instrumental variables:*					
Distance to nearest registry in km	5,157	4.849	4.147	0.036	28.6
Mother without document of identification	5,157	0.107	0.309	0	1
*Rest of controls:*					
Child is a girl	5,157	0.474	0.499	0	1
Card (seen)	5,157	0.709	0.454	0	1
Current age of the child (months)	5,157	30.03	17.54	0	59
Aged 0-2 months	5,157	0.02	0.141	0	1
Aged 3-6 months	5,157	0.09	0.291	0	1
Aged 7-12 months	5,157	0.100	0.301	0	1
Birth order	5,157	2.519	1.313	1	5
Born in hospital/health center	5,157	0.982	0.134	0	1
Mother's schooling in years	5,157	8.344	4.391	0	19
Mother works	5,157	0.291	0.454	0	1
One parent born abroad	5,157	0.042	0.201	0	1
Wealth index	5,157	2.333	1.302	1	5
Rural área	5,157	0.440	0.496	0	1
No water/electricity	5,157	0.033	0.179	0	1
Vaccinated in a campaign	5,157	0.354	0.478	0	1
Health center far away	5,157	0.296	0.456	0	1
Distance to nearest immunization center in km	5,157	2.360	2.375	0.008	18.4
Immunization center attends morning and afternoon	5,157	0.705	0.456	0	1

*Source:* Dominican Republic DHS (2007)

Our outcome variables capture both total uptake and timely vaccination. Total uptake is simply the total number of vaccines received by a child 0–59 months old. We also looked at uptake on individual vaccines. Several other dependent variables that seek to measure delays in vaccination were also constructed. The first variable on timely vaccination is the proportion of age-due vaccines actually delivered. This variable seeks to control not only for the number of vaccines received but also for those vaccines which are due in relation to the age of the child. This variable, in contrast with the total number of vaccines, does not take into account the vaccines outside the recommended age range but only those that had to be delivered within a certain time frame. The second variable is a dummy that measures complete vaccination at 7 months; another variable measures complete vaccination at 13 months. These ages were chosen because at 6 months a child ought to have received eight vaccines, and at 12 months ought to have received the full set of nine vaccines.

Table 1 also reports that around 30% of the households in the sample responded that the nearest health center is too far away. However, self-reported distances are prone to measurement error. Data were therefore collected on the exact location of immunization centers in the country and the distance from the cluster of households was calculated. The linear distance between health immunization centers and the cluster of households is only 2.4 kilometers on average, with a maximum of 18 kilometers. The Appendix: Figures 5 and 6) contains information on the location of each immunization center in the Dominican Republic in various provinces distinguished in colors and symbols and the frequency distribution of this linear GPS-measured distance. The different symbols and colors in the map of Figure A1 illustrate the location of immunization centers. We calculate the minimum distance to the nearest immunization center regardless of the location of the latter, as the parents are not bound or legally constrained to vaccinate their children in their home province. Very few households are located more than 10 kilometers from the nearest immunization center, and the immunization centers cover the entire national territory reasonably well. Moreover, around 70% of them offer services all day rather than only half a day.

- The dominican health system

The Dominican health system has both public and private sector components. In the public sector, immunization is provided free of charge to all, regardless of possession of identity documents. However, access to the private health care system and reimbursement by the state social security system requires proof of identity. Otherwise, Dominicans must pay out of pocket for private health services, including vaccines and shots.

Thus, the lack of documents may affect those children who are uninsured in remote areas where the state has little presence and who cannot afford the fees charged by private health care providers, reducing their access to immunization services. This hypothesis is tested by exploiting data on access to immunization centers, using self-reported perception of distance to health centers in the DHS^2^ and the GPS-measured linear distance to the nearest immunization center.

**Fig. 4:**
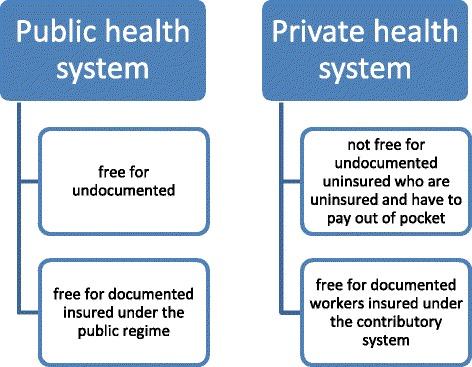
Health System Coverage in the Dominican Republic

**Fig. 5:**
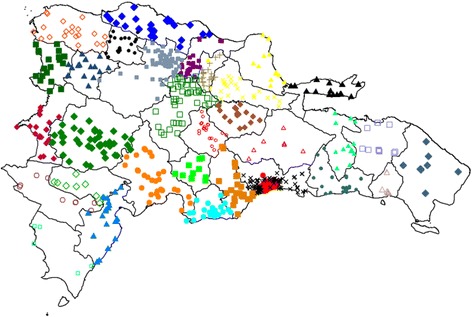
Location of immunization centers

**Fig. 6:**
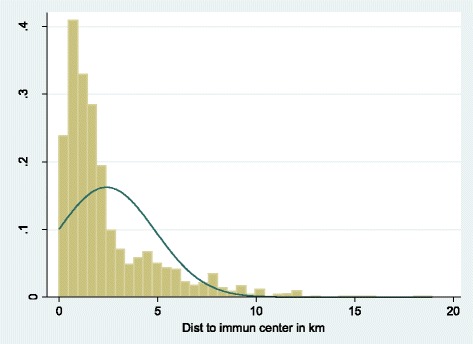
Frequency of distances to immunization centers

With regard to the legal framework, the two laws that define the structure of the health system in the Dominican Republican are the General Law on Health (*Ley 42-01*) and the Law on the Dominican Social Security System (*Ley 87-01*), both passed in 2001. They divide the health system into public and private providers. Figure 4 illustrates the provision of health care services in the Dominican Republic.

Article 3 of Law 42-01 grants Dominican citizens and foreign legal residents the right to health care. Law 87-01 also states that the Social Security System must serve all Dominicans and legal residents in the country without discrimination. In theory, these laws do not exclude undocumented people from public access to health care. In practice, the fact that 98% of women give birth in a health center or a hospital seems to corroborate what the laws state.

## Methods

This paper answers several questions. Does lacking a birth certificate reduce the number of vaccines delivered? Which vaccines are primarily affected? What are the potential mechanisms? Does birth under-registration reduce timely vaccination?

To address these questions, we ran several econometric models using the dependent variables described above on uptake and timely vaccination. The empirical strategy uses different limited dependent-variable models that relate vaccine outcomes to birth registration, children’s characteristics, mother’s characteristics, and other controls frequently used in the immunization literature.

Our empirical strategy takes into account potential endogeneity of our variable of interest, lack of a birth certificate. Vaccination may increase the incentive to register a child’s birth, generating reverse causation. In this case, the association between immunization and birth certificates would increase if vaccination increased the number of children with birth certificates. In such cases, children vaccinated will be more likely to have birth certificates. Nonetheless, the direction of the bias is jointly determined by the other factor that causes endogeneity. The association of omitted factors with vaccination and their correlation with birth registration is unknown. These omitted factors could include preference for health care services, in particular attitudes on vaccination, and birth registration. Thus, the bias of the coefficient is a priori unknown.

To address this potential endogeneity, we used two instrumental variables: (i) distance from the household cluster to the civil registry office, and (ii) whether or not the mother has an identity document *(cédula de identidad)*. Following Corbacho and Osorio [22] and Brito et al. [23], we use GPS-measured distance from the cluster of households to the civil registry office as an instrumental variable of whether or not a child has a birth certificate.^3^ There are several mechanisms through which distance to the registry office may decrease chances of a parent registering the child’s birth. An obvious one is transportation costs. Another may be lower access to information about the necessary steps and requirements to obtain a birth certificate. Corbacho and Osorio [22] also find that lack of legal identity of the mother explains the lack of birth certificates for her children, since it is one of the prerequisites to register a child’s birth. However, it is important to clarify that all children born on Dominican soil have the right to be Dominicans and receive identity documents regardless of their parents’ origin (the principle of jus solis), but in practice the requirement to present the parents’ documents of identification conflicts with this principle.

We explore the validity of these instrumental variables using a battery of econometric tests and by adding controls that might be correlated with the instrumental variables. For example, the distance to civil registry offices and the mother not having an identity document could be negatively correlated with the existence of health care services such as immunization centers. Thus, to our basic specification, we add as a control the distance to immunization centers to check for the stability of the coefficients in the presence of controls likely correlated with our instruments. After controlling for other determinants of vaccination, our two instrumental variables should not be expected to have an independent effect on vaccines, while being good predictors of birth registration. We also checked in Appendix: Table 8 if the instrumental variables were correlated with the outcomes of interest.

## Results

(i) First stage: correlation of birth certificates with distance and mother's ID

In the first stage, we explored the relationship between birth certificates, distance to civil registries, and mother’s identity document after controlling for other socioeconomic characteristics. Table 2 reports the marginal results for children aged 0-59 months of the regression:

**Table 2:** First Stage –Correlation of birth registration with instrumental variables

*Dependent variable:*	(2.1)	(2.2)	(2.3)	(2.4)
1 if child does not have birth certificate, 0 otherwise	OLS	PROBIT	OLS	PROBIT
Distance to nearest registry in km	0.010^***^	0.009^***^	0.011^***^	0.010^***^
	(0.002)	(0.002)	(0.002)	(0.002)
Mother without document of identification	0.376^***^	0.348^***^	0.376^***^	0.377^***^
	(0.017)	(0.026)	(0.018)	(0.028)
Aged 0-2 months	0.266^***^	0.327^***^	0.247^***^	0.314^***^
	(0.035)	(0.055)	(0.035)	(0.058)
Aged 3-6 months	0.084^***^	0.098^***^	0.068^***^	0.086^***^
	(0.017)	(0.022)	(0.017)	(0.023)
Aged 7-12 months	0.074^***^	0.086^***^	0.072^***^	0.088^***^
	(0.016)	(0.021)	(0.016)	(0.022)
Child is a girl	-0.018^*^	-0.017^*^	-0.015	-0.017
	(0.010)	(0.010)	(0.010)	(0.011)
Birth order	0.013^***^	0.012^***^	0.011^***^	0.011^**^
	(0.004)	(0.004)	(0.004)	(0.004)
Born in hospital/health center	-0.089^**^	-0.067	-0.060	-0.038
	(0.037)	(0.045)	(0.037)	(0.043)
Mother's schooling in years	-0.013^***^	-0.015^***^	-0.013^***^	-0.015^***^
	(0.001)	(0.001)	(0.001)	(0.002)
Mother works	-0.013	-0.024^**^	-0.017	-0.030^**^
	(0.011)	(0.012)	(0.011)	(0.012)
One parent born abroad	0.102^***^	0.075^**^	0.118^***^	0.102^***^
	(0.026)	(0.030)	(0.026)	(0.035)
Rural area	0.014	0.022	-0.001	0.014
	(0.013)	(0.014)	(0.015)	(0.017)
No water/electricity	0.018	-0.004	0.063^**^	0.027
	(0.029)	(0.027)	(0.031)	(0.034)
Health center far away	0.013	0.013	0.011	0.012
	(0.011)	(0.012)	(0.011)	(0.013)
Vaccinated in a campaign	-0.003	-0.005	-0.013	-0.014
	(0.011)	(0.012)	(0.011)	(0.012)
Dist to immun center in km	-0.004	-0.005	-0.003	-0.004
	(0.003)	(0.003)	(0.003)	(0.004)
Immun cent attends morning/afternoon	0.003	0.005	-0.000	-0.003
	(0.011)	(0.011)	(0.012)	(0.013)
Constant	0.177^***^		0.182^***^	
	(0.034)		(0.034)	
Household dummies	No	No	No	No
Province dummies	Yes	Yes	No	No
Municipality dummies	No	No	Yes	Yes
Observations	5157	5157	5157	5157
*R* ^2^	0.232		0.282	
Pseudo *R* ^2^		0.219		0.258

*Notes:* Marginal effects. Robust standard errors in parentheses. * *p* < 0.1, ** *p* < 0.05, *** *p* < 0.01

1 $$ {\displaystyle {NoBirthCert}_i^{*}}={\beta}_1+ mindis{t}_i{\beta}_2+ MotherI{D}_i{\beta}_3+{X}_i{\beta}_4+{\gamma}_j+{\varepsilon}_i $$

We used as the dependent variable whether or not the child had a birth certificate and, as predictors, the distance from each cluster of household where child i lives to the nearest civil registry office, a dummy variable indicating whether or not his/her mother lacked an identity document,controls *X*_*i,*_ and household dummies in some cases and province or municipality dummies in others. The Dominican Republic is divided into provinces, including the national district where the capital city is located. The next political subdivision is municipalities. We denote these political subdivisions as γ_i_ in our regressions.

Columns 1 through 4 show a strong and significant effect of both intended instrumental variables on the probability of not possessing a birth certificate. The marginal effects show that every kilometer is associated with an increase in the probability of a child not having a birth certificate of 0.01 percentage points. The mother possessing an identity document increases the probability of registering a child’s birth by at least 0.35 percentage points.

(ii) Second stage: impact of birth registration on immunization

The second stage of our analysis looked at the question: *What is the impact of birth registration on immunization?* The basic empirical specification is:

2 $$ V a c{c}_i={\beta}_0+ NoBirthCer{t}_i{\beta}_1+{X}_i{\beta}_2+{\gamma}_j+{\varepsilon}_i $$

where *Vacc*_*i*_ is any of the immunization variables listed in the summary statistics above for child *i*; *NoBirthCert*_*i*_ is a binary variable that indicates if child *i* does not have a birth certificate; *X*_i_ is a list of controls; γ_j_ are household dummies in some regressions and province or municipality dummies in others; and ε_i_ is the error of the equation. We used a combination of linear and non-linear models such as OLS, 2SLS and MLE^4^ models to account for endogeneity of birth certificates and for the fact that *Vacc*_*i*_ is a discrete variable.

The results of the regressions are reported in Table 3. There *Vacc*_*i*_ is the number of vaccines for children aged 0-59 months. MLE is a maximum likelihood estimator that derives a two-step estimator. In the first stage of the MLE, regression (1) is estimated, with *NoBirthCert*_i_^*^ being an unobserved probability. The only thing observed is when the child has a birth certificate, in which case the variable used in the first and second stage *NoBirthCert*_*i*_ is equal to 1 and is 0 otherwise. Marginal coefficients are reported as they are easier to interpret in the case of non-linear models.

**Table 3:** Effect of lack of birth certificate on number of vaccines (Age 0-59 months)

*Dependent variable*	(3.1)	(3.2)	(3.3)	(3.4)	(3.5)	(3.6)	(3.7)
Number of vaccines received by the child	OLS	OLS	2SLS	MLE	OLS	2SLS	MLE
Child without birth certificate	-0.276	-0.303^***^	-0.572^***^	-0.649^***^	-0.544^**^	-0.299^***^	-0.755^***^
	(0.214)	(0.059)	(0.204)	(0.152)	(0.224)	(0.060)	(0.156)
Card (seen)	0.016	0.217^***^	0.207^***^	0.214^***^	0.191^***^	0.237^***^	0.233^***^
	(0.291)	(0.047)	(0.047)	(0.043)	(0.050)	(0.047)	(0.042)
Aged 0-2 months	-4.950^***^	-6.040^***^	-5.956^***^	-5.935^***^	-5.855^***^	-5.992^***^	-5.852^***^
	(0.780)	(0.098)	(0.113)	(0.141)	(0.116)	(0.103)	(0.141)
Aged 3-6 months	-3.800^***^	-3.915^***^	-3.886^***^	-3.879^***^	-3.836^***^	-3.923^***^	-3.875^***^
	(0.282)	(0.076)	(0.078)	(0.068)	(0.083)	(0.077)	(0.067)
Aged 7-12 months	-1.304^***^	-1.269^***^	-1.244^***^	-1.237^***^	-1.194^***^	-1.278^***^	-1.235^***^
	(0.220)	(0.071)	(0.074)	(0.064)	(0.078)	(0.072)	(0.064)
Child is a girl	-0.085	0.026	0.022	0.020	-0.055	0.011	0.002
	(0.110)	(0.037)	(0.037)	(0.037)	(0.049)	(0.037)	(0.036)
Birth order	-0.016	-0.055^***^	-0.055^***^	-0.055^***^	-0.044^***^	-0.051^***^	-0.050^***^
	(0.087)	(0.017)	(0.017)	(0.015)	(0.017)	(0.017)	(0.015)
Born in hospital/health center	0.613	0.455^**^	0.427^**^	0.417^***^	0.495^**^	0.540^***^	0.494^***^
	(0.706)	(0.199)	(0.199)	(0.141)	(0.198)	(0.203)	(0.142)
Mother's schooling in years		0.026^***^	0.021^***^	0.019^***^	0.020^***^	0.027^***^	0.017^***^
		(0.005)	(0.006)	(0.006)	(0.006)	(0.005)	(0.006)
Mother Works		-0.006	-0.011	-0.011	-0.097	0.012	0.006
		(0.042)	(0.041)	(0.042)	(0.070)	(0.042)	(0.042)
One parent born abroad	0.119	-0.502^***^	-0.430^***^	-0.414^***^	-0.366^***^	-0.450^***^	-0.336^***^
	(0.180)	(0.134)	(0.141)	(0.102)	(0.141)	(0.133)	(0.103)
Rural area		0.025	0.039	0.042	-0.147	0.053	0.077
		(0.042)	(0.044)	(0.043)	(0.134)	(0.047)	(0.047)
No water/electricity		-0.696^***^	-0.679^***^	-0.676^***^	-0.693^***^	-0.787^***^	-0.768^***^
		(0.150)	(0.151)	(0.108)	(0.164)	(0.165)	(0.115)
Health center far away		-0.120^***^	-0.117^***^	-0.115^***^	-0.076	-0.124^***^	-0.118^***^
		(0.043)	(0.043)	(0.042)	(0.051)	(0.045)	(0.043)
Vaccinated in a campaign	0.221	0.059	0.056	0.057	0.117^**^	0.074^*^	0.072^*^
	(0.222)	(0.042)	(0.042)	(0.042)	(0.052)	(0.043)	(0.042)
Dist to immun center in km		-0.006	-0.005	-0.005	-0.018	-0.020	-0.019
		(0.010)	(0.010)	(0.010)	(0.012)	(0.012)	(0.012)
Immun cent attends morning/afternoon		0.050	0.051	0.051	-0.006	0.032	0.034
		(0.041)	(0.041)	(0.041)	(0.052)	(0.045)	(0.043)
Constant	7.307^***^	7.012^***^	7.098^***^	7.117^***^	6.288^***^	6.981^***^	7.118^***^
	(0.277)	(0.136)	(0.149)	(0.136)	(0.485)	(0.138)	(0.135)
Household dummies	Yes	No	No	No	No	No	No
Province dummies	No	Yes	Yes	Yes	No	No	No
Municipality dummies	No	No	No	No	Yes	Yes	Yes
Observations	5157	5157	5157	5157	5157	5157	5157
*R* ^2^	0.948	0.556	0.553		0.653	0.581	
*Under-identification test:*							
Kleibergen-Paap rk LM stat			220.9			220.4	
*Weak identification tests:*							
Cragg-Donald Wald F stat			255.4			244.3	
Kleibergen-Paap rk Wald F			154.5			147.5	
*Over identification test:*							
Hansen P-value			0.788				

*Notes:* Marginal effects. Robust standard errors in parentheses. * *p* < 0.1, ** *p* < 0.05, *** *p* < 0.01

The first OLS estimate in Table 3, with household dummies, shows that not having a birth certificate has no statistically significant effect on the number of vaccines. However, when we specified the age of the child in linear form, we obtained a significant estimate even with household dummies. Since having or not having a birth certificate is likely to be a household characteristic, we dropped household dummies to avoid problems with multicollinearity. As a result, the OLS regressions without household dummies in Table 3 then show coefficients around 0.5. These estimates suggest that the variation in the number of vaccines is associated with the lack of a birth certificate even after accounting for those unobservable factors correlated with not possessing a birth certificate. The instrumental variables are used in columns 3 and 4. The 2SLS specification in column 3 shows an effect of 0.57 fewer vaccines, but the MLE in column 4 shows a larger effect of nearly 0.65. The difference may be explained by the econometric specification because 2SLS treats the endogenous variable as linear, whereas the MLE treats it as binary.

With respect to other determinants, we find that children with their vaccination cards receive around 0.2 more vaccines than others. Those born in hospitals and health centers receive more vaccines than those born elsewhere, although the significance is not robust across the econometric specifications. This could be associated with the fact that it may be affecting only the BCG and HEPB vaccines, both given during the first two months of life, usually after birth. This is also consistent with the fact that these first vaccines have the highest uptake rates, as explored in further detail below.

One of the most robust findings in the literature on vaccines is that birth order affects the immunization of children in the same households. Older children receive more vaccines than their younger siblings, even after accounting for the difference attributable to their age. In fact, these results are statistically significant across most econometric specifications, except in the case when household dummies are introduced in column 1.

Mothers’ education increases vaccination, but the effect is small compared to other determinants. Children born of parents born abroad (the majority of whom are Haitian) receive at least 0.3 fewer vaccines than children whose parents are Dominicans. This could be due to myriad factors, such as language barrier, discrimination, or lack of awareness of the importance of vaccination. With regard to household characteristics, only two are significant: (i) lack of water or electricity; and (ii) wealth, but this characteristic is not robust across specifications, therefore we omitted it. It is also surprising that rural areas do not have lower immunization records than urban areas.

The next step was to add more controls in order to check the robustness of the results. The most immediate threat to the exclusion restriction is that parents who live far from health facilities may also live farther from civil registry offices and lack identity documents. If this threat to the exclusion restriction is real and the variables that capture access to immunization centers are unobserved, the coefficients in columns 3 and 4 would be biased despite using instrumental variables. Fortunately, we found information on access to health centers, mobile immunization campaigns, and location of permanent immunization centers.

The variables added were: i) *vaccinated in a campaign,* which measures if the child was vaccinated in any mobile vaccination campaign; ii) *health center far away,* which captures self-reported perception of distance to the health center; iii) *distance to nearest immunization center in km*, which involved collecting data on the location of more than 800 permanent immunization centers in the Dominican Republic; and iv) *immunization center open morning/afternoon.* To obtain data related to variables iii and iv, the address, hours of operation, and latitude and longitude of the immunization centers were collected. The results appear in columns 2 through 7 in Table 3. They still show the lack of a birth certificate has a negative and significant effect on vaccinations. Hence, the instrumental variables were robust to the addition of these crucial controls.

We used the standard battery of econometric tests performed to assess the validity of instruments. The tests at the bottom of Table 3 generally indicate that there are no reasons to cast doubt on the validity of the instrumental variables. This is so because they are sufficiently correlated with the endogenous variable and they are not correlated with the error term of regression (2). Column 7 is likely the most robust specification, with instrumental variables and dummies at the municipal level. This estimate suggests that lacking a birth certificate is associated with a reduction of 0.7 vaccines. Thus, not having this document seems to be an impediment to have complete vaccine coverage.

With regard to the new controls added to check for robustness of the basic specifications, we find that vaccination campaigns are strongly associated with immunization. Specifically, a child vaccinated in a mobile vaccination campaign receives 0.07 more vaccines than children vaccinated only at permanent immunization centers. The fact that the mother considers the health center far away is associated with a reduction in the number of vaccines by 0.12. Finally, the actual linear distance to the immunization center was not significant.

(iii) Effect on individual vaccines

The results so far established that lacking a birth certificate reduces the number of vaccines in children under 59 months of age. But, what can be said about the effect on each individual vaccine? We explore this in Table 4, which shows regressions for the BCG, HEPB, DTP1, DTP2, DTP3, OPV1, OPV2, OPV3 and MCV vaccines. The results presented come from IV-PROBIT specifications following the procedure described in Rivers and Vuong [36] to correct for endogeneity in a PROBIT model where the endogenous variable is also binary.

**Table 4:** Effect of Lack of Birth Certificate on Individual Vaccines

*Dependent variable:*	(4.1)	(4.2)	(4.3)	(4.4)	(4.5)	(4.6)	(4.7)	(4.8)	(4.9)
1 if child took vaccine, 0 otherwise	BCG	HEPB	DTP1	DTP2	DTP3	OPV1	OPV2	OPV3	MCV
Child without birth certificate	-0.031	-0.037	-0.131^**^	-0.117^*^	-0.088	-0.107^**^	-0.007	-0.135^*^	-0.299^***^
	(0.032)	(0.038)	(0.053)	(0.060)	(0.072)	(0.049)	(0.045)	(0.076)	(0.083)
Card (seen)	0.029^***^	-0.029^***^	0.027^***^	0.089^***^	0.175^***^	0.005	0.172^***^	0.426^***^	-0.143^***^
	(0.007)	(0.006)	(0.008)	(0.014)	(0.018)	(0.005)	(0.014)	(0.017)	(0.017)
Aged 0-2 months	0.010	0.026	-0.926^***^	-0.875^***^	-0.742^***^	-0.944^***^	-0.897^***^		-0.719^***^
	(0.012)	(0.017)	(0.023)	(0.009)	(0.008)	(0.024)	(0.007)		(0.013)
Aged 3-6 months	0.000	0.013	-0.268^***^	-0.646^***^	-0.772^***^	-0.166^***^	-0.634^***^	-0.793^***^	-0.776^***^
	(0.008)	(0.011)	(0.027)	(0.024)	(0.010)	(0.023)	(0.026)	(0.008)	(0.009)
Aged 7-12 months	0.013^**^	0.018^*^	-0.018	-0.103^***^	-0.268^***^	-0.007	-0.075^***^	-0.276^***^	-0.705^***^
	(0.005)	(0.010)	(0.013)	(0.023)	(0.027)	(0.009)	(0.022)	(0.028)	(0.014)
Child is a girl	-0.000	-0.004	0.005	0.006	0.004	-0.006	-0.003	-0.006	0.004
	(0.004)	(0.007)	(0.006)	(0.010)	(0.014)	(0.004)	(0.010)	(0.015)	(0.016)
Birth order	-0.002	0.001	-0.006^***^	-0.009^**^	-0.012^**^	-0.003	-0.008^**^	-0.009	-0.015^**^
	(0.002)	(0.003)	(0.002)	(0.004)	(0.006)	(0.002)	(0.004)	(0.006)	(0.006)
Born in hospital/health center	0.059^**^	0.096^**^	0.054^*^	0.075	0.130^**^	-0.003	-0.015	-0.011	0.168^***^
	(0.028)	(0.038)	(0.031)	(0.046)	(0.056)	(0.014)	(0.031)	(0.057)	(0.064)
Mother's schooling in years	0.001^*^	0.002^*^	0.001	0.004^**^	0.008^***^	-0.001	0.005^***^	0.008^***^	0.005^**^
	(0.001)	(0.001)	(0.001)	(0.002)	(0.002)	(0.001)	(0.002)	(0.002)	(0.003)
Mother works	-0.001	0.007	-0.000	-0.005	0.003	0.002	-0.002	0.016	-0.000
	(0.005)	(0.007)	(0.007)	(0.012)	(0.016)	(0.005)	(0.011)	(0.017)	(0.018)
One parent born abroad	-0.004	-0.050^**^	-0.010	-0.079^**^	-0.058	-0.001	-0.082^**^	-0.055	-0.041
	(0.012)	(0.023)	(0.017)	(0.036)	(0.042)	(0.011)	(0.035)	(0.043)	(0.048)
Rural area	0.013^**^	0.024^**^	0.014^*^	0.026^*^	0.018	0.006	0.022	0.015	0.035
	(0.006)	(0.010)	(0.009)	(0.015)	(0.021)	(0.006)	(0.014)	(0.022)	(0.023)
No water/electricity	-0.065^**^	-0.100^***^	-0.037	-0.112^***^	-0.248^***^	-0.001	-0.064^*^	-0.130^**^	-0.116^**^
	(0.028)	(0.035)	(0.024)	(0.043)	(0.049)	(0.012)	(0.036)	(0.054)	(0.057)
Health center far away	0.007	-0.004	-0.024^***^	-0.023^*^	-0.050^***^	-0.008	-0.004	-0.023	-0.028
	(0.004)	(0.008)	(0.008)	(0.012)	(0.017)	(0.005)	(0.011)	(0.017)	(0.019)
Vaccinated in a campaign	-0.011^**^	-0.014^*^	0.003	0.020^*^	0.018	-0.003	0.020^*^	0.026	0.027
	(0.005)	(0.008)	(0.007)	(0.012)	(0.016)	(0.005)	(0.011)	(0.016)	(0.018)
Dist to immun center in km	-0.002	-0.006^***^	0.003	-0.003	-0.006	0.000	-0.005^*^	-0.008^*^	-0.003
	(0.001)	(0.002)	(0.002)	(0.003)	(0.005)	(0.001)	(0.003)	(0.005)	(0.005)
Immun cent attends morning/afternoon	-0.006	0.009	0.006	0.017	-0.004	-0.000	-0.003	-0.031^*^	-0.008
	(0.005)	(0.008)	(0.007)	(0.013)	(0.017)	(0.005)	(0.012)	(0.018)	(0.018)
Observations	5157	5157	5157	5157	5157	5157	5157	5157	5157

Notes: All coefficients are marginal effects from regressions IV-PROBIT. Robust standard errors in parentheses. * *p* < 0.1, ** *p* < 0.05, *** *p* < 0.01. All regressions also include controls for municipality dummies

Three vaccines seem to be affected by the lack of a birth certificate: the DTP1, OPV1 and MCV. The coefficients were statistically significant also when we included dummy variables at the household level in OLS regressions (except for the MCV). Children without birth certificates have 13, 11, and 30 percentage points less likelihood of being vaccinated with DTP1, OPV1, and MCV, respectively. These results should be of great concern to national authorities and civil society because polio destroys motor neurons and causes muscle weakness, resulting in permanent physical damage. Diphtheria, tetanus, and pertussis are highly contagious and develop into epidemics quickly in large, populated areas. According to the WHO, measles is a leading cause of vaccine-preventable child mortality.

We also repeated the analysis using the sample of children with vaccination cards. The results are contained in the Appendix: Table 7. The results change but only slightly. For example, the impact of not having birth certificate on the number of vaccines changes from -0.76 to -0.69 and remains statistically significant. As for DTP1, OPV1 and MCV the effects are similar with the exception of the one for OPV1 which becomes not significant. The difference between these results and the ones obtained with the whole sample could be attributed to unobservable attributes that are correlated with the possession of a vaccination card, and to the reduction of degrees of freedom because only 70% of the children have these cards.

## Discussion

- Why does the lack of a birth certificate affect vaccination?

One mechanism of transmission could be related to the need to prove a child’s age in order to receive a specific vaccine. Given that in the Dominican Republic there is a schedule of vaccinations that recommends that vaccines be given at a particular age, not having proof of age-appropriateness could be one channel of transmission. The vaccination schedule is based on the fact that the immune system’s response is optimal at the recommended age. Undocumented immigrants are also afraid to enter public services facilities, where proof of documentation is needed, in order to avoid problems with their immigration status. Therefore, it is highly likely that mothers whose children lack identity documents avoid visiting immunization centers.

(b) Does the lack of a birth certificate produce delays in vaccination?

We also examined the effect of lack of birth certificates on delays in immunization delivery. The results are reported in Table 5. The sample is composed of children with vaccination cards because these cards are the source of information about the vaccination date. The regressions control for all of the determinants that appear in the tables above.

**Table 5:** Effect of Lack of Birth Certificate on Timely Vaccination

	(5.1)	(5.2)	(5.3)
	Proportion of age-due vaccines (children aged 0-59 months)	Up to date vaccinations at 7 months (children aged >7 months)	Up to date vaccinations at 12 months (children aged > 12 months)
Child without birth certificate	-0.108^**^	-0.020	-0.265^***^
	(0.054)	(0.093)	(0.080)
Card (seen)	2.181^***^		
	(0.088)		
Aged 0-2 months	0.121^***^		
	(0.046)		
Aged 3-6 months	-0.164^***^		
	(0.015)		
Aged 7-12 months	-0.033^*^	-0.040	
	(0.019)	(0.027)	
Child is a girl	0.023^**^	0.032^*^	0.016
	(0.012)	(0.018)	(0.021)
Birth order	-0.021^***^	-0.023^***^	-0.018^**^
	(0.005)	(0.008)	(0.009)
Born in hospital/health center	0.031	-0.037	-0.041
	(0.051)	(0.090)	(0.099)
Mother's schooling in years	0.002	0.001	-0.004
	(0.002)	(0.003)	(0.004)
Mother works	0.002	0.015	0.020
	(0.014)	(0.021)	(0.024)
One parent born abroad	-0.078^**^	-0.123^***^	-0.121^*^
	(0.035)	(0.047)	(0.064)
Rural area	-0.009	-0.018	-0.003
	(0.015)	(0.027)	(0.031)
No water/electricity	-0.087^***^	-0.132^**^	-0.151^**^
	(0.033)	(0.058)	(0.070)
Health center far away	-0.002	-0.021	0.016
	(0.013)	(0.022)	(0.025)
Vaccinated in a campaign	-0.050^***^	-0.051^**^	-0.052^**^
	(0.014)	(0.020)	(0.022)
Dist to immun center in km	-0.000	-0.002	-0.007
	(0.003)	(0.006)	(0.007)
Immun cent attends morning/afternoon	0.033^**^	0.051^**^	0.022
	(0.013)	(0.021)	(0.024)
Constant	-1.551^***^		
	(0.087)		
Observations	3478	3066	2594

*Notes:* Coefficients are marginal effects from regressions IV-TOBIT for column (5.1) and from regressions IV-PROBIT for columns (5.2) and (5.3). Robust standard errors in parentheses. All regressions include municipality dummies

* *p* < 0.1, ** *p* < 0.05, *** *p* < 0.01

Among children without birth certificates, the proportion of age-due vaccines decreases by about 10% according to the estimate in column 1. Up to date vaccinations at 7 months is not affected by the lack of a birth certificate. However, the probability of having complete, up-to-date vaccinations at 12 months is reduced by 27 percentage points if the child lacks a birth certificate, as suggested by the IVPROBIT regression in column 3.

## Conclusions

Healthy children do better in school, and as adults they are more productive at work. Health care at an early age, including immunization, is thus a crucial component of long-term economic prosperity. However, while much research on the socioeconomic characteristics of infant vaccination has been conducted, nothing has been said about the effect of the lack of legal identity on access to health services. This is the first study that aims to quantify a causal impact of the lack of a birth certificate on infant vaccination. The Dominican Republic is a highly relevant case because it is one of the few countries in Latin America and the Caribbean where under-registration birth is considerable.

We found that children without birth certificates are behind by 0.7 vaccines compared to those with birth certificates. In addition, the probability of vaccination with DTP1, OPV1, and MCV is reduced by 8, 7, and 19 percentage points respectively. Moreover, timely vaccination is less likely to occur when a child lacks a birth certificate. The proportion of age-due vaccines for children of a given age is reduced by 10 percent, and the probability of vaccination in due time at 12 months of age is reduced by 23 percentage points.

These findings have important policy implications. Around 98% children are born in hospitals and health centers and more than 90% receive at least the first two vaccines, BCG and HEPB. Given that health services have better coverage and far more outreach activities than civil registries, there is an opportunity to integrate their work in order to reduce the percentage of children without birth certificates and increase immunization rates.

## Appendix

**Table 6:** Permanent Immunization Centers

Province	Open Morning	Open Morning/Afternoon	Number
Azua	11	26	37
Bahoruco	3	9	12
Barahona	8	17	25
Dajabon	5	14	19
Distrito Nacional	26	70	96
Duarte	7	17	24
El Seybo	3	9	12
Elias Pina	5	14	19
Espalliat	9	20	29
Hato Mayor	6	12	18
Hermanas Mirabal	6	18	24
Independencia	2	7	9
La Altagracia	2	7	9
La Romana	3	5	8
La Vega	12	32	44
Maria Trinidad Sanchez	4	10	14
Monseñor Noel	7	17	24
Monte Christi	5	15	20
Monte Plata	2	5	7
Pedernales	2	3	5
Peravia	6	18	24
Puerto Plata	9	22	31
Samana	5	12	17
San Cristobal	10	25	35
San Jose Ocoa	4	11	15
San Juan de la Maguan	13	37	50
San Pedro de Macoris	8	22	30
Sanchez Ramirez	8	22	30
Santiago	21	52	73
Santiago Rodriguez	5	12	17
Santo Domingo	23	57	80
Valverde	5	15	20
Total	245	632	877

*Source*: Health Ministry of the Dominican Republic

**Table 7:** Effect of Lack of Birth Certificate on Vaccination using Data from Vaccination Cards

	(A2.1)	(A2.2)	(A2.3)	(A2.4)
Dependent variable	MLE2 number of vaccines	IVPROBIT DTP1	IVPROBIT OPV1	IVPROBIT MCV
Child without birth certificate	-0.694^***^	-0.292^***^	-0.077	-0.278^**^
	(0.180)	(0.107)	(0.054)	(0.114)
Aged 0-2 months	-6.155^***^		-0.986^***^	-0.600^***^
	(0.147)		(0.004)	(0.017)
Aged 3-6 months	-4.111^***^	-0.283^***^	-0.175^***^	-0.744^***^
	(0.069)	(0.031)	(0.028)	(0.010)
Aged 7-12 months	-1.370^***^	-0.021	-0.010	-0.694^***^
	(0.066)	(0.014)	(0.010)	(0.015)
Child is a girl	0.023	0.001	-0.009^**^	0.018
	(0.041)	(0.007)	(0.005)	(0.024)
Birth order	-0.070^***^	-0.009^***^	-0.003	-0.038^***^
	(0.018)	(0.003)	(0.002)	(0.010)
Born in hospital/health center	0.303	0.048	-0.008	0.135
	(0.194)	(0.044)	(0.012)	(0.102)
Mother's schooling in years	0.003	-0.003^**^	-0.001	-0.001
	(0.007)	(0.001)	(0.001)	(0.004)
Mother works	-0.010	0.010	0.000	-0.001
	(0.048)	(0.007)	(0.005)	(0.027)
One parent born abroad	-0.566^***^	-0.009	-0.013	-0.057
	(0.125)	(0.019)	(0.014)	(0.074)
Rural area	0.047	0.007	-0.007	0.009
	(0.060)	(0.010)	(0.007)	(0.033)
No water/electricity	-0.576^***^	0.006	0.006	-0.060
	(0.146)	(0.019)	(0.009)	(0.088)
Health center far away	-0.047	-0.002	-0.000	-0.031
	(0.049)	(0.008)	(0.005)	(0.028)
Vaccinated in a campaign	-0.059	-0.008	-0.007	-0.024
	(0.049)	(0.009)	(0.006)	(0.026)
Dist to immun center in km	-0.001	0.004^*^	0.002	0.001
	(0.013)	(0.002)	(0.001)	(0.008)
Immun cent attends morning/afternoon	0.027	0.003	-0.005	-0.006
	(0.049)	(0.008)	(0.005)	(0.028)
Household dummies	No	No	No	No
Province dummies	No	No	No	No
Municipality dummies	Yes	Yes	Yes	Yes
Observations	3654	3654	3654	3654

*Notes:* Marginal effects; Standard errors in parentheses. * *p* < 0.1, ** *p* < 0.05, *** *p* < 0.01

**Table 8:** Effect of Instrumental Variables on Vaccinations

	(A3.1)	(A3.2)	(A3.3)	(A3.4)
Dependent variable:	Number of vaccines	DTP1	DTP3	MCV
Child without birth certificate	-0.263^***^	-0.028^***^	-0.039^**^	-0.042^***^
	(0.062)	(0.011)	(0.016)	(0.014)
Distance to nearest registry in km	-0.017	-0.001	-0.002	-0.003
	(0.010)	(0.002)	(0.002)	(0.002)
Mother without birth certificate	-0.115	-0.024	-0.011	-0.030
	(0.085)	(0.020)	(0.022)	(0.020)
Card (seen)	0.233^***^	-0.002	0.062^***^	-0.121^***^
	(0.047)	(0.007)	(0.013)	(0.012)
Aged 0-2 months	-5.984^***^	-0.912^***^	-0.816^***^	-0.780^***^
	(0.104)	(0.023)	(0.019)	(0.022)
Aged 3-6 months	-3.918^***^	-0.240^***^	-0.790^***^	-0.784^***^
	(0.077)	(0.021)	(0.014)	(0.013)
Aged 7-12 months	-1.275^***^	-0.023^**^	-0.212^***^	-0.703^***^
	(0.072)	(0.011)	(0.022)	(0.017)
Child is a girl	0.012	0.003	0.008	0.008
	(0.037)	(0.006)	(0.010)	(0.009)
Birth order	-0.054^***^	-0.008^***^	-0.010^**^	-0.011^***^
	(0.017)	(0.003)	(0.005)	(0.004)
Born in hospital/health center	0.524^***^	0.075^**^	0.123^**^	0.092^**^
	(0.202)	(0.037)	(0.048)	(0.044)
Mother's schooling in years	0.024^***^	0.001	0.005^***^	0.003^**^
	(0.005)	(0.001)	(0.001)	(0.001)
Mother works	0.012	0.001	0.006	0.001
	(0.042)	(0.007)	(0.012)	(0.010)
One parent born abroad	-0.408^***^	-0.038^*^	-0.052^*^	-0.043
	(0.137)	(0.023)	(0.031)	(0.031)
Rural area	0.135^**^	0.014	0.017	0.015
	(0.059)	(0.010)	(0.016)	(0.014)
No water/electricity	-0.746^***^	-0.048^*^	-0.216^***^	-0.068^*^
	(0.165)	(0.028)	(0.039)	(0.037)
Health center far away	-0.118^***^	-0.023^***^	-0.035^***^	-0.016
	(0.045)	(0.008)	(0.012)	(0.011)
Vaccinated in a campaign	0.075^*^	0.005	0.012	0.014
	(0.043)	(0.006)	(0.012)	(0.011)
Dist to immun center in km	-0.009	0.002	-0.005	0.002
	(0.013)	(0.002)	(0.004)	(0.003)
Immun cent attends morning/afternoon	0.033	0.011	0.010	-0.003
	(0.045)	(0.008)	(0.012)	(0.011)
Household dummies	No	No	No	No
Province dummies	No	No	No	No
Municipality dummies	Yes	Yes	Yes	Yes
Observations	5157	5157	5157	5157
*R* ^2^	0.582	0.343	0.393	0.539

*Notes:* Coefficients from OLS regressions. Robust standard errors in parentheses. * *p* < 0.1, ** *p* < 0.05, *** *p* < 0.01

## Footnotes

1 The DHS was not designed to study legal identity issues. Fortunately for the Dominican Republic, the 2006 ENHOGAR household survey asked about birth certificates. This provided a secondary and independent source of data to cross-check the accuracy of birth registration rates in the DHS. We were able to confirm that birth registration rates in both datasets coincide, being 22 percent for children under the age of 5.

2 Health centers may not coincide with immunization centers, but they may serve as a proxy for the actual location of immunization centers, as they generally include a unit devoted to immunizations.

3 The DHS contains a random error in the position of the cluster of households. This is done to protect the confidentiality of the household members. See http://dhsprogram.com/faq.cfm for more details.

4 We used the treatreg command in STATA.
